# 25 years of neurocognitive aging theories: What have we learned?

**DOI:** 10.3389/fnagi.2022.1002096

**Published:** 2022-09-23

**Authors:** Ian M. McDonough, Sara A. Nolin, Kristina M. Visscher

**Affiliations:** ^1^Department of Psychology, Alabama Research Institute on Aging, The University of Alabama, Tuscaloosa, AL, United States; ^2^Department of Neurobiology, The University of Alabama at Birmingham, Birmingham, AL, United States

**Keywords:** neurocognition, fMRI, aging, older adults, theory, review, maintenance, compensation

## Abstract

The past 25 years have provided a rich discovery of at least four fundamental patterns that represent structural and functional brain aging across multiple cognitive domains. Of the many potential patterns of brain aging, few are ever examined simultaneously in a given study, leading one to question their mutual exclusivity. Moreover, more studies are emerging that note failures to replicate some brain aging patterns, thereby questioning the universality and prevalence of these patterns. Although some attempts have been made to create unifying theories incorporating many of these age-related brain patterns, we propose that the field’s understanding of the aging brain has been hindered due to a large number of influential models with little crosstalk between them. We briefly review these brain patterns, the influential domain-general theories of neurocognitive aging that attempt to explain them, and provide examples of recent challenges to these theories. Lastly, we elaborate on improvements that can be made to lead the field to more comprehensive and robust models of neurocognitive aging.

## Introduction

**FIGURE 1:**
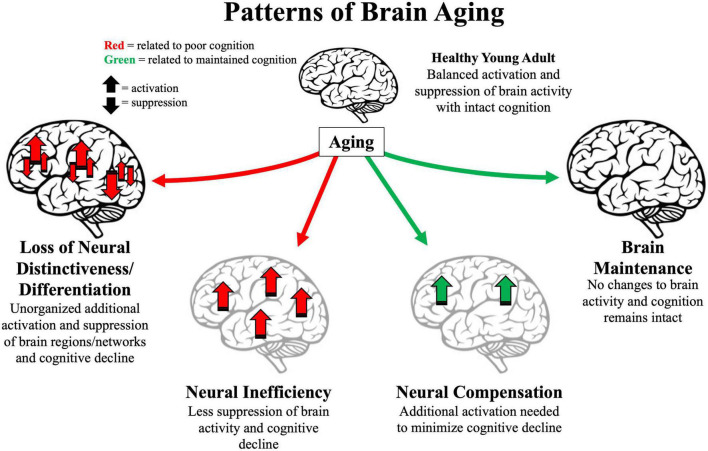
Illustration of four major patterns associated with older age. Arrows represent activation or suppression of brain activity associated with maintained (green) or poor (red) cognitive performance. Light gray brains represent brain structure degradation. Location of arrows represent primary brain areas patterns implicated in brain patterns. Loss of neural distinctiveness/differentiation **(left)** is characterized by decreased difference of brain signals to different perceived categories or loss of modularity of specific brain networks. Neural inefficiency **(lower left)** is characterized by non-beneficial increases in brain activity. Neural compensation **(lower right)** is characterized by beneficial increases in frontoparietal regions. Brain maintenance **(right)** is characterized by some older adults’ brain structure and function as in young adulthood **(top)**.

Understanding human brain aging became much more feasible following the advent of non-invasive-neuroimaging methods. The following 25 years yielded many observed patterns of brain aging and many neurocognitive theories of aging proposed to explain those patterns. In this review, we summarize early theories that explained domain-general declines in cognition and more recent patterns of brain aging that have been subsequently observed (Figure 1 and Table 1). These patterns have been used as evidence toward various modern neurocognitive aging theories. We provide examples of recent challenges to the generalizability of previously observed patterns, implicating a need for more robust theories. We then discuss ways to build on these inconsistencies to advance theories of neurocognitive aging.

**TABLE 1:** Summary of key aspects of the four major brain aging patterns.

	Loss of neural distinctiveness /Differentiation	Brain maintenance	Neural compensation	Neural inefficiency
Relation with PFC/PPC	Decreased network segregation in frontal networks	Youthlike or longitudinally maintained levels of structure and function	Increases in brain activity and connectivity; decreases in brain structure	Increases in brain activity
Relation with other brain regions	Decreased network segregation in non-frontal networks; Loss of brain signal selectivity in sensory and motor cortices	Youthlike or longitudinally maintained levels of structure and function	Increases in activity in new/secondary brain regions	Increases in brain activity
Relation with cognition	Lower distinctiveness/differentiation is associated with lower cognition	The more youthlike or maintained longitudinally maintained, the better cognition	Increases in brain activity in PFC/PPC associated with better cognition	Increases in brain activity is not related to cognition or is related to lower cognition
Mechanisms	Loss of dopaminergic neurons, loss of GABAergic neurons	Loss of neurotransmitter systems; elevated neuropathological lesions; genetic variants; lifestyle factors; environmental factors	Decreases in brain activity (e.g., sensory cortex, medial temporal lobe, default mode regions); decreases in brain structure; elevated neuropathological lesions; genetic variants; lifestyle factors; environmental factors	Loss of GABAergic neurons; lower white matter integrity
Key theories/articles	Li et al., 2001; Reuter-Lorenz and Park, 2014;Koen and Rugg, 2019	Nyberg et al., 2012; Reuter-Lorenz and Park, 2014	Greenwood, 2007; Davis et al., 2008; Reuter-Lorenz and Cappell, 2008; Reuter-Lorenz and Park, 2014; Cabeza et al., 2018; Spreng and Turner, 2019	Reuter-Lorenz et al., 2001; Logan et al., 2002

For clarity, we distinguish between “brain aging patterns” and “neurocognitive aging theories” such that multiple theories may explain a brain aging pattern (i.e., observed phenomenon) and some brain aging patterns may not have an explicit theory tied to them but may have theoretical mechanisms suggested when first described. We refer to theories as an overarching framework that goes beyond the observed phenomenon and provides mechanisms, related constructs, and predictions for a brain aging pattern. *Italics* are used when describing theories to differentiate theoretical inferences from observed data.

### Early neurocognitive aging theories

Building upon notions of cognitive-brain deficits from neuropsychological and lesion approaches, one early neurocognitive theory of aging based on structural magnetic resonance imaging (MRI) was the *Frontal Lobe Hypothesis of Aging.* This hypothesis emphasized early shrinkage of the prefrontal cortex (PFC) in middle-age purportedly responsible for multiple cognitive deficits (e.g., West, 1996) and was concordant with white matter disconnection theories to explain generalized cognitive slowing with age (Cerella, 1990; Salthouse, 1992). Relatedly, the *Last-In-First-Out Hypothesis* proposed that the last brain regions to myelinate were the most vulnerable to degradation, pointing again to the PFC because of its early white matter degradation (Reisberg et al., 1999).

Early functional neuroimaging techniques (Xenon^133^ inhalation or positron emission tomography, PET) showed consistency with structural MRI scans, evidencing either stable or declining patterns of cerebral blood flow in older age (West, 1996). These patterns of functional under-recruitment often were attributed to a decline in neural resources (e.g., Logan et al., 2002). However, a few PET studies suggested a pattern of age-related *hyper*activity in the PFC and sensory cortex (e.g., Grady et al., 1992; Cabeza et al., 1997). Subsequent studies, including those using functional MRI (fMRI), continued to show mixed patterns of increases and decreases in brain activity during various cognitive tasks that were not easily explained by early structural theories.

## Influential patterns of brain aging

### Loss of neural distinctiveness/differentiation

Dedifferentiation refers to the process of becoming less distinct. A neural dedifferentiation/distinctiveness pattern refers to brain activity becoming less selective and discretely organized with age (Koen and Rugg, 2019). For example, brain regions that selectively activate in response to a specific stimulus, such as the ventral visual cortex for visual objects and faces, do not activate as selectively in older adults (Park et al., 2004). Dedifferentiation has been proposed to be caused by declines in the brain’s primary inhibitory neurotransmitter gamma-aminobutyric acid (GABA) (Lalwani et al., 2019; Chamberlain et al., 2021) and the loss of dopamine receptors in PFC and striatal regions that help regulate attention to specific details (e.g., Li et al., 2001).

This pattern also has been extended to the declines in differentiation of brain networks (Chan et al., 2014; Cassady et al., 2020; Pedersen et al., 2021). In young adults, brain networks show distinct patterns of synchronous temporal fluctuations. Portions of these networks also are recruited when engaged in a task but are less distinguishable in older adults (Grady et al., 2016). These declines in differentiation (or desegregation) are associated with poorer cognition both in cross-sectional (Park et al., 2010; Goh, 2011) and longitudinal studies (Ng et al., 2016; Malagurski et al., 2020).

### Brain maintenance

Brain maintenance encompasses many of the patterns of brain degradation into a unified theme (Nyberg et al., 2012). Brain maintenance emphasizes that not all older adults exhibit the same patterns of aging; some show an absence of typical age-related patterns, representing “preserved” brain structure and function from young adulthood. Similarly, the *Scaffolding Theory of Aging and Cognition* (STAC) proposed that chronological age is not the driver of brain alterations throughout the lifespan (Park and Reuter-Lorenz, 2009). Rather, neural insults (e.g., brain shrinkage, dopamine depletion, white matter degradation) could occur at any age and these neural insults cause alterations in brain functioning. The revised theory (*STAC-r*) incorporates life-course experiences more explicitly (e.g., stress, fitness, education) that affect brain degradation or preservation (Reuter-Lorenz and Park, 2014). Thus, a middle-aged adult with many negative life-course experiences might have a brain resembling a typical older adult. Brain aging patterns that deviate from a maintained or youthlike state often are related to poorer cognition.

### Neural compensation due to brain degradation

In contrast to deficit perspectives of aging, a neural compensation pattern is when aging is related to increases in neural activity, particularly in the PFC, thought to benefit cognition. Such age-related increases have been observed in bilateral PFC activity during demanding tasks (e.g., Cabeza, 2002; Reuter-Lorenz and Cappell, 2008), in the PFC with lower brain activity in sensory cortex (*Posterior-to-Anterior Shift in Aging* or *PASA*; Davis et al., 2008), in the PFC and the medial temporal lobes (MTL) with decreases in nearby white matter (Daselaar et al., 2015), and in the coupling of PFC regions with the MTL (Daselaar et al., 2006; Dennis et al., 2008) or default mode regions; Spreng and Turner, 2019). Given the connectivity between the PFC and the lateral parietal cortex (Dosenbach et al., 2008; Vincent et al., 2008), such compensatory increases have been extended to the parietal lobe (e.g., Nagel et al., 2009; Huang et al., 2012).

The observed increases in frontoparietal activity have been considered a direct response to age-related structural or functional degradations in the PFC, MTL, posterior/sensory cortex, or an inability to modulate the default mode network (e.g., Li et al., 2001; Greenwood, 2007; Davis et al., 2008; Park and Reuter-Lorenz, 2009; Spreng and Turner, 2019). This compensatory neural response should minimize the impact of brain degradation on cognition, which we dubbed the *Atrophy-Compensation Hypothesis* (McDonough and Madan, 2021). This neural compensation should occur in nearby or contralateral brain regions to the sites of brain atrophy (e.g., Cabeza et al., 2002; Greenwood, 2007; Reuter-Lorenz and Cappell, 2008). In *STAC*, neural compensation was termed scaffolding and referred to the activation of secondary brain regions/networks (primarily in the PFC) that differed from the original (young) brain regions used to engage in a task (Park and Reuter-Lorenz, 2009). Scaffolding is similar to cognitive reserve in the context of cognitive reserve (Stern, 2002). Recently, the idea of neural compensation has been refined into different forms depending on when and where in the brain they occur (Cabeza et al., 2018). Regardless of the different forms, each generally supports cognition.

### Neural inefficiency

Brain activity increases also could be due to an inefficient neural system that either does not contribute to current cognitive processes (non-selective activity) or is detrimental to ongoing processes (Reuter-Lorenz et al., 2001; Logan et al., 2002). A neural inefficiency pattern is when an increase in brain activity relates to poorer cognition with aging. Such activity increases might stem from deficits in inhibitory neural circuitry (Lalwani et al., 2019) or lower white matter integrity (Bennett and Rypma, 2013). Accordingly, some brain regions do not show increased activity because they are actively suppressed in young adults, but when the “brakes” are released, increases in brain activity can be revealed. This perspective also predicts that negative relationships exist between white matter integrity and brain activity in older adults because the failures to inhibit brain activity might stem from white matter disconnections (Bennett and Rypma, 2013). Some support for neural inefficiency comes from studies showing that cognitive training decreases brain activity (Lustig et al., 2009; McDonough et al., 2015; Nguyen et al., 2019; Ross et al., 2019).

## Challenges to neurocognitive theories of aging

### ¿Qué pasa con PASA?

Morcom and Henson (2018) sought to replicate a neural compensation pattern predicted by *PASA* in a large adult lifespan sample across two different cognitive tasks. Using a model-based decoding approach, they also tested whether the predicted age-related increases in PFC (*via* a multivariate pattern) carried additional information about cognitive performance. Although older age was associated with increases in PFC activity in both tasks, concomitant age-related decreases in sensory cortex were not found, failing to support *PASA*. In fact, the visual-perception task showed age-related increases in brain activity in sensory cortex. Moreover, the patterns of PFC activity carried less information about cognitive performance as age increased, did not predict cognitive performance beyond that found in the sensory cortex, and revealed strong evidence in favor of the null hypothesis using Bayes factor scores. Thus, this study failed to find evidence for *PASA* or evidence for compensation in the PFC but was consistent with a pattern of neural inefficiency.

### Revisiting the atrophy-compensation hypothesis

Given the proposal that brain degradation should be associated with increased brain activity (regardless of whether it is compensatory), supporting evidence is surprisingly sparse in healthy aging samples (Brassen et al., 2009; Pudas et al., 2013). Using a sensitive marker of brain degradation (fractal dimensionality), McDonough and Madan (2021) directly tested whether brain degradation in one hemisphere was associated with increases in PFC activity in nearby or contralateral brain regions during successful memory encoding and retrieval. Bayes factor scores revealed moderate to strong evidence across PFC regions supporting no relationship between brain degradation and brain activity in either task in the PFC, challenging the *Atrophy-Compensation Hypothesis*.

## Toward better neurocognitive aging theories

These counter examples suggest that some neurocognitive aging theories need revising if the fundamental patterns of data on which they are partly based are unreliable. More theoretical progress might be made if changes are made in (a) how theory testing (or lack thereof) is conducted and (b) how analyses are conducted to reveal brain patterns, especially if drawing from heterogeneous samples. Both changes have important implications for the inferences that can be made from brain patterns.

### The impact of pre-registration

We currently do not have a good grasp as to the exclusivity or co-existence of the four major patterns of brain aging outlined here. Better adjudication between neurocognitive aging theories could shed light onto how these patterns relate to one another or represent distinct facets of the aging process. However, most findings from individual neurocognitive aging studies either support one of the many existing patterns of brain aging or were not designed to test major theories of neurocognitive aging. Furthermore, most brain studies on aging have (understandably) not been pre-registered. Without pre-registrations, we do not know which theories originally guided a study, whether the original hypotheses were abandoned after viewing the results, or whether results were interpreted within existing theories *post-hoc*. For example, one might design a study to investigate age-related bilateral PFC activity but if that pattern was not found, then the framing might shift to one consistent with brain maintenance. These practices are akin to a theoretical “file drawer” effect in which some theories continue to be critically unexamined, and no clear consensus exists for a leading theory of neurocognitive aging. Thus, one first step is to document what brain aging pattern is predicted as guided by a theory in a pre-registration. Doing so will help establish the ease (or difficulty) of finding a given brain aging pattern as relevant to a new experimental design and context.

### Testing competing models

One also could explicitly test competing predictions between different neurocognitive theories of aging. Indeed, some theories make predictions that are either mutually exclusive or somewhat incompatible. For example, the *Atrophy-Compensation Hypothesis* predicts an inverse relationship between structural integrity and functional activation (McDonough and Madan, 2021). Theories with this hypothesis as a component might predict that if structural decline occurs in sensory cortex, then brain activity should increase in nearby or contralateral brain regions. While structural declines do occur in posterior regions (Salat et al., 2004; Fjell et al., 2009; Storsve et al., 2014), other theories (e.g., *PASA*) or hypotheses derived from other brain patterns (e.g., neural dedifferentiation) propose decreases in posterior brain activity, which would lead to positive rather negative associations. Similarly, one also could test which of multiple mechanisms best explains age-related increases in PFC activity: PFC structural degradation in the opposite hemisphere, lower posterior brain activity, or neural dedifferentiation. By engaging in model comparisons or designing paradigms to test competing accounts, the field can move beyond providing support for an existing theory and start ruling out or modifying existing theories that do not explain patterns of brain aging as well as others (Popper, 1963).

### Bridging seemingly different brain patterns

These analytic strategies may not be appropriate if multiple patterns of brain aging are due to the same underlying cause or are different manifestations of the same theory. For example, lower sensory cortex activity might be one manifestation of neural dedifferentiation in sensory cortex. Both decreased brain activity and neural dedifferentiation in sensory cortex might be caused by underlying degradations of white matter in nearby tracts (Rieck et al., 2020). Similarly, network desegregation might simply be a different method of testing neural dedifferentiation. Indeed, both network desegregation and neural dedifferentiation are significantly correlated (albeit weakly) with one another (Cassady et al., 2020). A third factor might cause both patterns independently or one pattern may cause the other. One candidate is the loss of inhibition, which has also been linked to neural inefficiency. However, more critical tests are needed either linking or dissociating these patterns into current neurocognitive theories.

### Multiple brain aging patterns in different groups of older adults

In the previous sections, we assumed that older adults (as a whole) exhibited homogeneous patterns of brain aging in a given sample. However, some brain aging patterns might be found in only subsets of aging adults, and thus can be better understood through individual differences. As documented in *STAC*, older adults who are more physically fit, engage in more cognitive stimulation, or have fewer genetic risk factors have been proposed to employ neural scaffolds more effectively (Park and Reuter-Lorenz, 2009). Relatedly, brain maintenance acknowledges that variability in genetic and lifestyle factors help explain why some older adults can maintain a healthy (youthlike) brain (Nyberg et al., 2012). A recent study provides an illustration of how one might explore multiple brain aging patterns among subgroups of older adults. Chen et al. (2022) first separated middle-aged and older adults into “successful” and “average” cognitive agers using longitudinal changes in cognition. Successful cognitive agers exhibited similar levels of brain activity as young adults in sensory cortex using a subsequent-memory contrast, supporting the brain maintenance pattern. In contrast, the average agers showed reduced subsequent-memory activation compared to both young adults and successful agers in both PFC and sensory cortex. This perspective emphasizes that different people can show different patterns of brain aging. While we acknowledge that many studies have successfully shown individual differences, the direction and location of brain activation often are difficult to predict and such predictions have not been made concrete in many neurocognitive aging theories.

### Multiple brain aging patterns in the same brain

A more overlooked and conceptually distinct idea is that multiple patterns of brain aging might coexist in each person (e.g., Logan et al., 2002; Cassady et al., 2020). For example, an individual might exhibit a loss of differentiation in one part of the brain (e.g., visual cortex) and exhibit brain maintenance in a different part of the brain (e.g., the PFC). In this case, a person exhibits at least two brain aging patterns. However, we do not often characterize people at the individual level. Large, diverse samples and person-centered clustering methods might be a practical approach that can characterize common brain patterns in subgroups. The study by Chen et al. (2022) continues to be illustrative. Not only did successful agers exhibit a “maintained brain” in sensory cortex, but they also showed greater subsequent-memory activation in the PFC than both average cognitive agers and young adults, consistent with a neural compensation pattern. If we tentatively assume that the successful aging group was a homogeneous subgroup, then inspecting each person’s brain activity should show two patterns (brain maintenance and neural compensation) but in different regions of the brain.

### Theories need to prospectively predict aging brain patterns

As elaborated by West (1996), a valuable theory of neurocognitive aging should be able to accurately predict (a) when age-related deficits should be observed, (b) when age-related differences are not observed (age-invariance), and (c) identify the specificity or generality of the sources of the effects. Although West (1996) was articulating the relationships between neuropsychological tests and brain integrity, the same holds for modern neurocognitive aging theories. How precisely can we predict which brain regions will show functional increases, decreases, or remain unchanged with age? Can we predict the mechanism of brain aging patterns? Could those predictions be made precisely enough to be pre-registered?

### Recommendations for future research

• Use existing neurocognitive theories to inform directionally (i.e., increases vs. decreases) and regionally specific hypotheses.
• Pre-register theories and hypotheses motivating the study.
• Create analyses that test competing or alternative models or mechanisms.
• Use sufficiently large and diverse sample sizes to test for heterogenous patterns of brain aging.

## Conclusion

The last 25 years of cognitive aging research has resulted in a rich body of aging brain patterns and multiple neurocognitive aging theories. However, understanding of the aging brain has been hindered due to little crosstalk between those theories. Recently, even the underlying brain patterns that gave rise to some of those theories have been questioned, providing an impetus to critically inspect existing neurocognitive aging theories. By better understanding (a) which aspects of theories overlap with one another, (b) which aspects of a given theory survive direct tests, and (c) the conditions under which some brain patterns might occur, existing theories can be falsified (or modified), leading to more comprehensive and robust models of neurocognitive aging that make generalizable predictions about the aging brain.

## Author contributions

IM: conceptualization, visualization, writing – original draft preparation, and writing – review and editing. SN and KV: visualization, writing – original draft preparation, and writing – review and editing. All authors contributed to the article and approved the submitted version.
